# A novel *Pectobacterium brasiliense*-infecting phage from Egypt with biocontrol potential against soft rot in vegetables

**DOI:** 10.3389/fmicb.2025.1621267

**Published:** 2025-09-25

**Authors:** Kamel M. Elhalag, Abdelmonim Ali Ahmad, Mohsen Mohamed Elsharkawy, Qi Huang, Mohamed A. Nasr-Eldin

**Affiliations:** ^1^Department of Bacterial Diseases Research, Plant Pathology Research Institute, Agricultural Research Center, Giza, Egypt; ^2^Department of Plant Pathology, Faculty of Agriculture, Minia University, El-Minia, Egypt; ^3^Department of Agricultural Botany, Faculty of Agriculture, Kafrelsheikh University, Kafr El-Sheikh, Egypt; ^4^Floral and Nursery Plants Research Unit, United States National Arboretum, Agricultural Research Service, United States Department of Agriculture, Beltsville, MD, United States; ^5^Department of Botany and Microbiology, Faculty of Science, Benha University, Benha, Egypt

**Keywords:** bacteriophage, soft rot *Pectobacteriaceae*, genome analysis, stability, soft rot suppression

## Abstract

*Pectobacterium brasiliense* causes soft rot in many economically important crops, including vegetables and ornamentals, leading to significant yield losses. Traditional antibiotics, bactericides, and antimicrobial agents face limitations such as bioaccumulation on plants and the emergence of microbial resistance. Bacteriophages (phages) offer a promising alternative for effective control of a variety of phytopathogens. In this study, we isolated and characterized a virulent phage as a potential biocontrol agent against *P. brasiliense*. The phage was designated as PbrM1EGY, as it specifically targets only tested strains of *P. brasiliense* (Pbr) and is the first *Myoviridae* phage (M1) isolated from Egypt (EGY). The phage has an icosahedral head measuring 46.3 ± 3.9 nm and a long, contractile tail of 99.9 ± 3.9 nm. Phage PbrM1EGY exhibited relative stability across a wide range of environmental conditions, including pH (3 to 11), temperatures (4–50°C), NaCl concentrations (1–15%), and ultraviolet light exposure (1–15 min). It takes about 50 min for PbrM1EGY to complete its lytic cycle with a latent period of approximately 20 min and an average burst size of 749 ± 40 PFU per infected cell. At MOIs of 0.01, 0.1 and 1, phage PbrM1EGY significantly reduced the *in vitro* growth of *P. brasiliense* compared to the bacterial control without phage treatment. When tested *in planta*, the phage effectively prevented the development of soft rot symptoms in pepper and cucumber fruits, carrot roots, and potato tubers, while significantly reducing tissue maceration in onion bulbs. Our findings suggest that phage PbrM1EGY has great potential as a biocontrol agent against soft rot disease caused by *P. brasiliense* in vegetable crops, including pepper, cucumber, carrot, onion and potato.

## 1 Introduction

*Pectobacterium* is one of the top 10 notorious plant pathogenic bacteria, responsible for causing soft rot in potatoes, ornamental plants, and various other vegetable crops (Mansfield et al., 2012) both in the field and during storage (Charkowski, 2018). Key virulence factors of *Pectobacterium* include plant cell wall-degrading enzymes (PCWDEs) and their secretion systems, production of toxins and plant hormones, motility, and the modulation of plant responses to infection (Gijsegem et al., 2021). Soft rot pathogens secrete a variety of PCWDEs during pathogenesis, including pectinases, cellulases, proteases, and xylanases. These enzymes are the primary pathogenic factors responsible for soft rot, as they break down plant cell walls and, in conjunction with other enzymes, ultimately lead to tissue decay (Marquez-Villavicencio et al., 2011). The disease symptoms of pectinolytic *Pectobacterium* infection include slow leaf wilt, stem rot, and soft rot. Some infected potato tubers may appear healthy in the field but rot during storage, leading to significant losses in both yield and quality (Zhang et al., 2016; Carstens et al., 2019). Potato soft rot is mainly transmitted by seed potato systems, rain, cutting tools, and insects (Czajkowski et al., 2009; Rossmann et al., 2018). Management of potato soft rot is challenging due to the widespread nature of the pathogen, lack of resistance in commercial cultivars, and the absence of effective disease control agents (Czajkowski et al., 2011).

Among the *Pectobacteriaceae*, *P. brasiliense* is considered as one of the most pathogenic species. This bacterium is widely dispersed beyond horticultural crops and has a broad host range within them. It is pathogenic both in the field, where it causes soft rot and blackleg in potatoes, and in storage, where it leads to soft rot in other vegetables (Oulghazi et al., 2021). *P. brasiliense*, previously known as *P. carotovorum subsp. brasiliensis*, primarily infects potatoes and other vegetables and ornamentals (Portier et al., 2019). After being discovered in Brazil in 2004, the bacterium was later found in the United States (Ma et al., 2007). Since then, it has been identified in association with a variety of plants, causing symptoms in many regions around the world. *P. brasiliense* was first reported in Egypt in 2013 (Behiry, 2013; Ashmawy et al., 2015) and has since been identified in many regions of Europe, Asia, Africa, Australia, and both North and South America (Van der Wolf et al., 2017). The bacterium has adapted to a wide range of temperatures and host species, making it a significant cause of crop losses in China, South Africa, Brazil, the Netherlands, Switzerland, and the UK (Meng et al., 2017; van der Wolf et al., 2017). Symptoms caused by *P. brasiliense* are indistinguishable from those of other soft rot *Pectobacterium* and *Dickeya* that are known to cause blackleg, tuber soft rot, stem wilt and stem rot (Kim et al., 2009). As a result, it is impossible to identify *P. brasiliense* based solely on field and laboratory symptoms. *P. brasiliense* also causes plant maceration and water-soaked lesions, leading to tissue collapse and plant wilting and death. Because *P. brasiliense* spreads more easily through plant vascular systems and can induce infection at lower inoculum levels than other soft rot bacteria in the genera *Erwinia* and *Dickeya*, it is considered more aggressive than other soft rot bacteria (USDA, 2015).

Unfortunately, there is no evidence of curative measures and varietal resistance (in cultivated potatoes) against this group of bacteria. As a result, farmers rely on seed certification, exclusion, and sanitation to mitigate its worst effects. *P. brasiliense* has a broad host range and is found in both tropical (e.g., Brazil) and temperate regions (Europe and Canada). *P. brasiliense* prefers humid subtropical conditions, making it extremely virulent across a range of temperatures from cool to warm (Du Raan et al., 2016), with an optimal growth temperature of 31–32°C, and has been reported as one of the most aggressive *Pectobacterium* species (Bellieny-Rabelo et al., 2018). The bacterium poses a major threat to cultivated vegetables, flowers, and even field crops. There are no curative measures for soft rot disease caused by *P. brasiliense*, making disease control particularly challenging. Traditionally, the control of bacterial diseases in crops has relied on chemical pesticides and cultural practices. While these methods have shown some effectiveness, they also present significant challenges. Excessive use of pesticides raises concerns about their environmental impact, the development of resistance in target pathogens, and the potential harm to non-target organisms. Utilizing nature’s defenses through biological control offers a promising alternative for managing bacterial pathogens.

Bacteriophages (phages) are viruses that specifically infect and kill disease-causing bacteria. They offer several advantages as alternative antimicrobial agents in agriculture compared to conventional chemical biocides: they are harmlessness to humans and beneficial bacteria, have relatively lower development and production costs than antibiotics, and can self-dose (Matsuzaki et al., 2005; Meaden and Koskella, 2013). Several therapeutic uses of phages for the biocontrol of *Pectobacterium* have been studied. Phages PP1, DU_PP13B, f A38, f 41, PcaP1EGY and PcaP2EGY were characterized and successfully protected lettuce and potato tubers from *P. carotovorum* infections (Lim et al., 2013; Lee et al., 2017; Smolarska et al., 2018; Elhalag et al., 2024). Some of the studies have assessed these isolated phages as biocontrol agents in more detail. So far, however, only a limited number of phages have been found to specifically infect *P. brasiliense* species, including phages PP99 and PP101 isolated from Russia (Lukianova et al., 2020) and seven phages isolated from fields in Denmark (Pedersen et al., 2024).

In this study, we report the characterization and genomic analysis of the novel phage PbrM1EGY isolated in Egypt that specifically targets *P. brasiliense*. We also evaluated the suitability of the phage as a biocontrol to suppress maceration symptoms caused by *P. brasiliense* in various parts of tested vegetable plants (*in planta* assay), based on its physical properties, lytic activity, growth curve, and host range.

## 2 Materials and methods

### 2.1 *Pectobacterium brasiliense* strain used in this study

A virulent strain of *P. brasiliense*, KMBR1, was used to isolate a *P. brasiliense*-specific phage and to study the biological and physical characteristics of the phage. The bacterial strain was isolated from potato tubers collected from a potato field at Zefta, Gharbia Governorate, Egypt during the winter growing season of 2023, showing typical symptoms of soft rot and black leg diseases. Logan’s and King’s media (Fahy and Hayward, 1983; King et al., 1954) were used for sub-culturing of the bacterium before its identity was confirmed. Genomic DNA of the bacterium was extracted using QIAamp DNA Kit (QIAGEN, UK). It was then amplified by conventional PCR using primers BR1f (5’-GCG TGC CGG GTT TAT GAC CT-3′) and L1R (5’-CAR GGC ATC CAC CCGT-3′) that are species-specific for *P. brasiliense* with an amplicon size of 322 bp based on the method of Duarte et al. (2004). The PCR amplification program was 95°C for 5 min, followed by 30 amplification cycles of denaturing at 94°C for 30 s, annealing at 62°C for 45 s, and extension at 72°C for 90 s, and ended with a final extension at 72°C for 7 min. The PCR product was visualized in 1.5% agarose gel by staining with ethidium bromide. The 16S rDNA sequencing of *P. brasiliense* KMBR1 was conducted at the Bacterial Diseases Research Department, Agricultural Research Center, Egypt, using universal primers 27f (5’-AGAGTTTGATCMTGGCTCAG-3′) and 1492r (5’-TACCTTGTTACGACTT-3′) based on Frank et al. (2008). The 16S rRNA sequence was analyzed by the 8-capillary genetic analyzer (Applied Biosystem, Scotland, UK) using Applied Biosystem’s Big-Dye Terminator Cycle Sequencing Kit. The 16S rDNA sequence of *P. brasiliense* strain KMBR1 was deposited in GenBank under the accession number of PP484882. *P. brasiliense* KMBR1 was confirmed to be virulent using a potato tuber maceration assay (Czajkowski et al., 2014) since it produced typical maceration symptoms on potato tubers.

### 2.2 Phage isolation

A phage was isolated from clay soil collected from Gharbia Governorate, Egypt using *P. brasiliense* strain KMBR1 as a bacterial host. Briefly, 5 g of soil was suspended in 10 mL of distilled water and vigorously shaken for 1 h at room temperature to release the phage, followed by centrifugation at 5000 rpm for 10 min. Five milliliters of the supernatant was then mixed with 50 mL of King’s B broth inoculated with the overnight culture of *P. brasiliense* KMBR1 to a final concentration of 10^8^ CFU/mL. The mixture was incubated overnight at 28°C, before centrifugation at 5000 rpm for 10 min. The supernatant was filtered through a 0.45-μm membrane filter (Cobbeter Corporation, China) to exclude bacterial cells and stored as a phage stock at 4°C for further study. The phage stock was serially diluted 10-fold and was used for plaque-forming and spot assays to identify the presence of the phage. The assays were conducted with *P. brasiliense* strain KMBR1 as the bacterial host in King’s B medium plates containing 1.5% agar overlaid with King’s B medium soft agar (0.45%) as described by Mihara et al. (2016). The plates were incubated overnight at 28°C for the formation of phage plaques. A single plaque was removed, placed in SM buffer (50 mM Tris–HCl at pH 7.5, 100 mM NaCl, 10 mM MgSO_4_, and 0.01% gelatin; Mihara et al., 2016), vortexed, and filtered through a 0.45 μm membrane. The process was sequentially repeated at least three times in order to obtain a pure phage. The phage titer was determined by the double-layer agar (DLA) method (Adams, 1959) and was expressed as plaque forming unit (PFU) per mL (PFU/mL).

### 2.3 Phage propagation and concentration

When the culture of *P. brasiliense* KMBR1 reached an optical density at 600 nm (OD_600_) of 0.4, the phage stock mentioned above was diluted and added at a multiplicity of infection (MOI) of 0.01–1.0. After growing at 28°C for 12–24 h, bacterial cells were removed by centrifugation at 5000 rpm for 10 min at 4°C. The supernatant was filtered through a 0.45 μm membrane, serially diluted, and plaque-forming assay was carried out to determine the phage titer. To make concentrated phage stock, phage particles in 45 mL of phage lysate (10^11^ PFU/mL) were pelleted by ultracentrifugation at 12,000 rpm for 3 h at 4°C and suspended in 300 μL of SM buffer based on Elhalag et al. (2018). The high titer phage stock was stored at 4°C for further study.

### 2.4 Determination of phage morphology

The purified phage was morphologically characterized by transmission electron microscopy (TEM) analysis as described by Kim and Ryu (2011). Briefly, 5 μL of the concentrated phage stock was placed on carbon-coated copper grids and negatively stained with 2% aqueous uranyl acetate (pH 4.0). Phage particles were observed under TEM (JEOL JEM-2100, Japan Electron Optics Laboratory Co., Ltd) at the Nano Tech Company, Giza, Egypt. The phage was classified morphologically using International Committee on Taxonomy of Viruses (ICTV)’s classification scheme (King et al., 2011).

### 2.5 Determination of phage host range and specificity

The host range of the phage was determined by both spot and plaque assays as described by Elhalag et al. (2024) using a collection of 12 *Pectobacterium* strains including *P. brasiliense*, *P. atrosepticum*, *P. carotovorum*, and *P. aroidearum*, as well as other 15 plant pathogenic or antagonistic bacteria (Table 1). These bacteria were isolated in Egypt (Elhalag et al., 2015, 2020, 2024; Farag et al., 2017) and maintained at the culture collection of the Bacterial Diseases Research Department, Plant Pathology Research Institute, Giza, Egypt. For phage spot assay, the bacterial lawn was prepared as described by Elhalag et al. (2024). Briefly, 300 μL of each of the tested bacterial cells (10^8^ CFU/mL, 24 h-old) was mixed into 3 mL of King’s B soft agar (0.4% agar) and poured on top of King’s B medium plates containing 1.5% agar. After the top agar was solidified for 30 min, 10 μL of the phage lysate were spotted on top the double layered King’s B plates and dried for 20 min at room temperature. The plates were incubated for 12 h at 28°C and recorded for the presence or absence of clearing zones as indications of host or non-host of the phage.

**Table 1:** Specificity of phage PbrM1EGY on pathogenic and antagonistic bacteria.

Bacterium		Strain^a^	Spot test*	Plaque assay*
Pathogenic	*Pectobacterium brasiliense*	KMBR1	+	+
		1HBR2	+	+
		KMM	+	+
	*P. atrosepticum*	MH3c	−	−
		MH2	−	−
		Fel2	−	−
		MH8	−	−
	*P. carotovorum*	GH1	−	−
		GH2	−	−
		100H	−	−
		GH5	−	−
	*P. aroidearum*	P.ash	−	−
	*Robbsia andropogonis* (*Burkholderia andropogonis*)	Ba	−	−
	*Ralstonia solanacearum* (Phylotype IIA sequevar 1)	K6	−	−
		K9	−	−
		K10	−	−
	*Rhizobium radiobacter* (*Agrobacterium tumefaciens*)	AGt	−	−
	*Erwinia amylovora*	Ea	−	−
Antagonistic	*Pseudomonas japonica*	447	−	−
	*Pseudomonas putida*	600B	−	−
	*Pseudomonas fluorescense*	200B	−	−
	*Enterobacter aerogenes*	114	−	−
	*Bacillus thuringiensis*	400B	−	−
	*Bacillus subtilis*	500B	−	−
	*Serratia marcescens*	100B	−	−
	*Pseudomonas aeruginosa*	177	−	−
	*Stenotrophomonas maltophilia*	300B	−	−

^a^All bacterial strains used in this study were isolated in Egypt (Elhalag et al., 2015, 2020, 2024; Farag et al., 2017) and maintained at the culture collection of the Bacterial Diseases Research Department, Plant Pathology Research Institute, Giza, Egypt. * +: the phage was able to form plaques. −: the phage was unable to form plaques.

### 2.6 Single-step growth experiment

Single-step growth experiment was performed as previously described by Yamada et al. (2010) with some modifications. *P. brasiliense* strain KMBR1 cells (OD_600_ of 0.3) were harvested by centrifugation at 8000 × *g* for 15 min at 4°C and resuspended in a final volume of 10 mL of fresh King’s B medium (approximately 1 × 10^8^ CFU/mL). The phage was added at a MOI of 1 and allowed to adsorb for 10 min at 28°C. After centrifugation at 8000 × *g* for 15 min at 28°C, the pellet was resuspended in initial volume of King’s B medium, and serial dilutions were prepared in a final volume of 10 mL. The cells were incubated at 28°C. An aliquot was taken every 10 min for 2 h and the phage titers were determined using double-layered King’s B agar plates.

### 2.7 Determination of phage stability under different temperature and pH conditions

The thermal stability test was conducted according to Jurczak-Kurek et al. (2016) with slight modifications. Briefly, 100 μL of the purified phage lysate (3 x 10^11^FU/mL) was added to 900 μL of SM buffer and the mixture was incubated for 1 h at 4, 30, 40, 50, 60, 70, 80, and 90°C, respectively. After incubation, the phage titers were determined using the DLA method. The experiment was repeated three times.

The pH stability of the phage was tested as described by Verma et al. (2009) with minor adjustments. Sterile King’s B broth with different pH values (2, 3, 5, 7, 9, 11 and 12) was prepared. One hundred microliters of the purified phage lysate were mixed with 900 μL of the pH-adjusted broth and incubated at 28°C for 1 h. The phage titers were determined as described above. The experiment was repeated three times.

### 2.8 Determination of phage stability under different NaCl concentration and UV exposure conditions

To study the effect of salt concentrations on phage stability, the phage was tested under different NaCl concentrations ranging from 1 to 25%. To do that, phage aliquots were added to different concentrations of NaCl solution with a pH of 7 and incubated for 1 h at room temperature. After incubation, the phage was enumerated using the plaque assay with *P. brasiliense* strain KMBR1 as the host bacterium (Elhalag et al., 2024). The experiment was repeated three times.

Phage stability under UV radiation was tested based on the method of Ramirez et al. (2018) with simple modifications. Briefly, 1 mL of the purified phage lysate (3× 10^11^ PFU/mL) was irradiated for 1, 5, 10, 15, 20 and 25 min, respectively, under the UV light (*λ* = 365 nm; 320 mW/m2). The number of viable phage was determined after each UV light exposure using the plaque assay. The experiment was repeated three times.

### 2.9 Phage DNA extraction, sequencing and genome analysis

Phage lysate with at least 10^9^ PFU/mL was used for DNA extraction using the phenol extraction method as described by Sambrook and Russell (2001). DNA purity and concentration were determined using the NanoDrop ND-1000 spectrophotometer (Thermo Fisher Scientific, Wilmington, United States) based on Karumidze et al. (2013). Phage DNA was sequenced commercially on an Illumina MiSeq with 2 × 300 bp reads and the genome sequence was assembled using Spades v3.12 by AmpSeq (Gaithersburg, Maryland, USA). Open reading frames (ORFs) exceeding 30 amino acids (aa) were identified through the software described by Besemer (2001). Potential function for each predicted ORF was determined with homology searches using Basic Local Alignment Search Tool for nucleotide sequence (BLASTn) and protein BLAST (BLASTp) (Altschul et al., 1997). An e-value threshold of e−4 or lower was established to qualify two proteins as a match. Complete genome sequence of the phage was analyzed against the GenBank database via BLASTn in order to identify closely related phages.

### 2.10 Phage effect on growth of its bacterial host at different MOI

After an overnight culture of *P. brasiliense* strain KMBR1 was inoculated into King’s B broth and grew at 28°C to the early exponential phase (OD_600_ of 0.4), purified phage stock was added at a MOI of 0.01, 0.1 and 1.0, respectively. King’s B broth without the addition of the phage was designated as a control. The OD_600_ was measured every hour for 12 h. The experiment was repeated three times.

### 2.11 Biocontrol of soft rot in pepper, cucumber, carrot, onion, and potato

To investigate the biocontrol potential of the phage against soft rot disease, pepper and cucumber fruits, carrot roots, onion bulbs, and potato tubers were surface sterilized with 70% ethanol, rinsed with sterilized distilled water, and air dried under a laminar flow hood. Two wells (5-mm in diameter/well) were made using a sterile cork borer in pepper, cucumber, and carrot, with a distance of at least 1 cm between the two wells, while one well was made in onion bulbs and potato tubers. Each well was inoculated with 40 μL of *P. brasiliense* KMBR1 (10^8^ CFU/mL), followed by the addition of 40 μL of the phage stock at a MOI of 1,000. Inoculated potato tubers were covered with the cut-out portion of the tuber and sealed with a paraffin wax (He et al., 2023). Inoculated pepper, cucumber, carrot, and onion were covered with sterile cotton balls soaked with sterile water, wrapped in plastic and kept at room temperature. Vegetable parts inoculated only with *P. brasiliense* KMBR1 (10^8^ CFU/mL) are designated as pathogen controls, and those inoculated only with sterilized distilled water (SDW) as negative controls. The inoculated vegetables were transferred to an incubator maintained at room temperature and inspected daily for the appearance of soft rot symptoms. Three days after incubation, the diameter of the rotting tissues surrounding the wells was determined, and the progress of tissue maceration was tracked for a period of 2 weeks. The diameters were averaged for each treatment after the experiment was repeated twice with five replicates for each vegetable in each treatment.

### 2.12 Statistical analysis

Data for thermal and pH stability of the phage, along with the performance of the phage under different NaCl concentration and UV exposure conditions, as well as the *in vitro* growth of *Pectobacterium brasiliense* strains 12 h post-treatment with phage PbrM1EGY at MOIs of 0.01, 0.1, and 1.0, were statistically evaluated using one-way analysis of variance (ANOVA). This analysis was performed with a freely accessible online statistical tool.^1^ To facilitate multiple comparisons of group means, Tukey’s Honest Significant Difference (HSD) test, included within the software, was employed.

Additionally, to assess the effect of phage PbrM1EGY treatment on disease severity, separate t-tests were conducted in Microsoft Excel for each vegetable type to compare disease severity caused by *P. brasiliense* strains with and without phage treatment. A *p*-value of less than 0.05 was considered statistically significant.

## 3 Results

### 3.1 Isolation of *Pectobacterium brasiliense*–infecting phage

**Figure 1:**
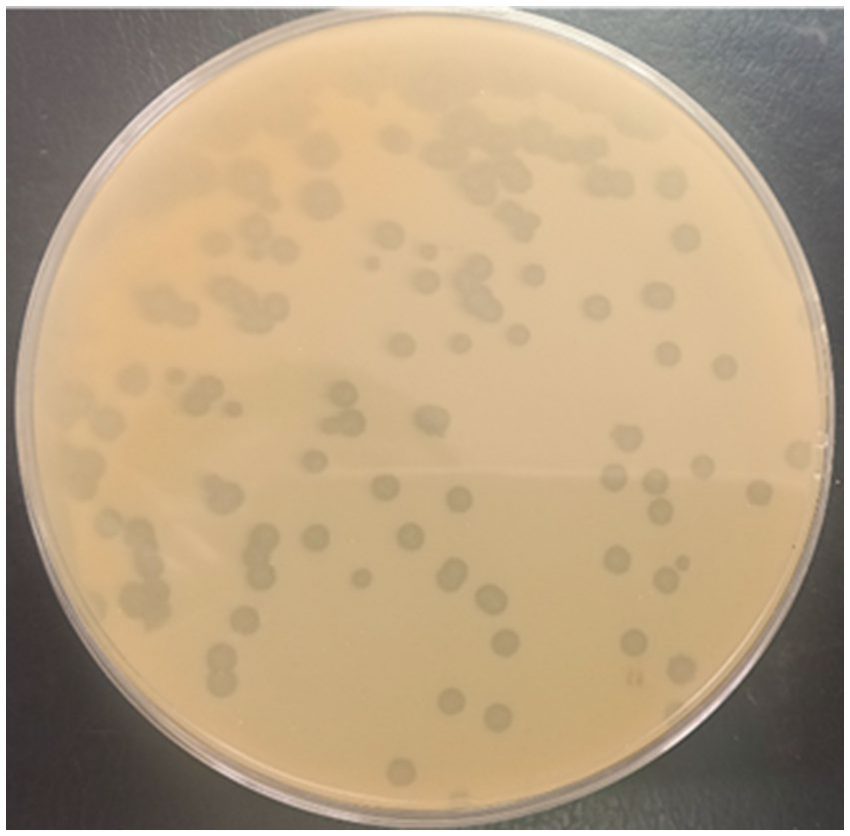
Appearance of plaques produced by phage PbrM1EGY on a double layered King’s B medium plate using *P. brasiliense* as a bacterial host.

A Phage designated as PbrM1EGY was isolated in Egypt using *P. brasiliense* strain KMBR1 as a bacterial host from soil previously infested by the bacterium. The purified phage displayed remarkable lytic activity against *P. brasiliense* as revealed by both spot and plaque assays and produced medium size of plaques approximately 2–4 mm in diameter (Figure 1).

### 3.2 Host range of phage PbrM1EGY

Phage PbrM1EGY was found to be specific only to *P. brasiliense*. The phage infected all tested strains of *P. brasiliense* isolated in Egypt (Table 1). However, it did not exhibit any lytic activity against other tested *Pectobacterium* spp. or against the other pathogenic or antagonistic bacterial species included in the study, suggesting that its activity is specific only to *P. brasiliense* (Table 1).

### 3.3 Morphology of phage PbrM1EGY

**Figure 2:**
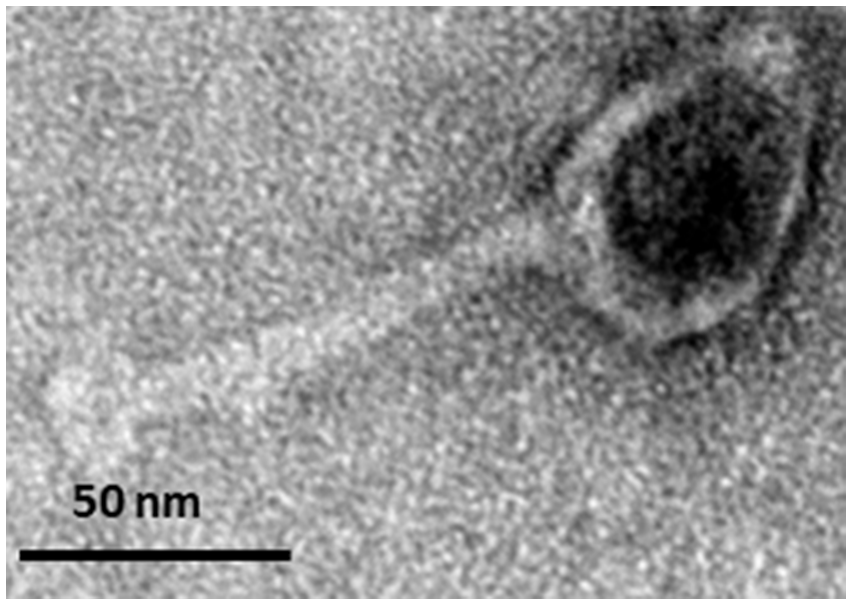
Transmission electron micrograph displaying the morphology of negatively stained Egyptian *P. brasiliense*-infecting phage PbrM1EGY. The scale is indicated.

Phage PbrM1EGY has an icosahedral head of 46.3 ± 3.9 nm in diameter (Figure 2). It also has a contractile tail of 99.9 ± 3.9 nm in length. Its morphology is typical of a myovirus (Figure 2).

### 3.4 Infection cycle of phage PbrM1EGY

**Figure 3:**
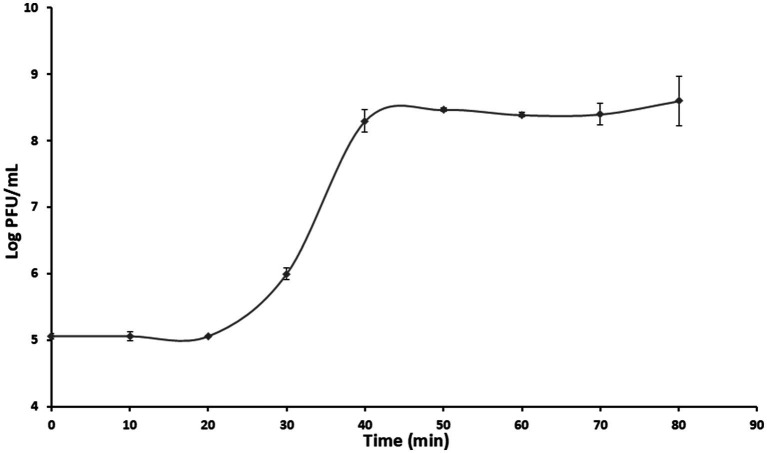
One-step growth curve of phage PbrM1EGY at MOI of 1. Means at each time points are based on two separate experiments, each containing three replicates. Bars represent standard deviation.

The phage had a latent period of approximately 20 min and its mean burst size was approximately 749 ± 40 PFU per infected cell (Figure 3).

### 3.5 Stability of phage PbrM1EGY under different pH and temperature conditions

**Figure 4:**
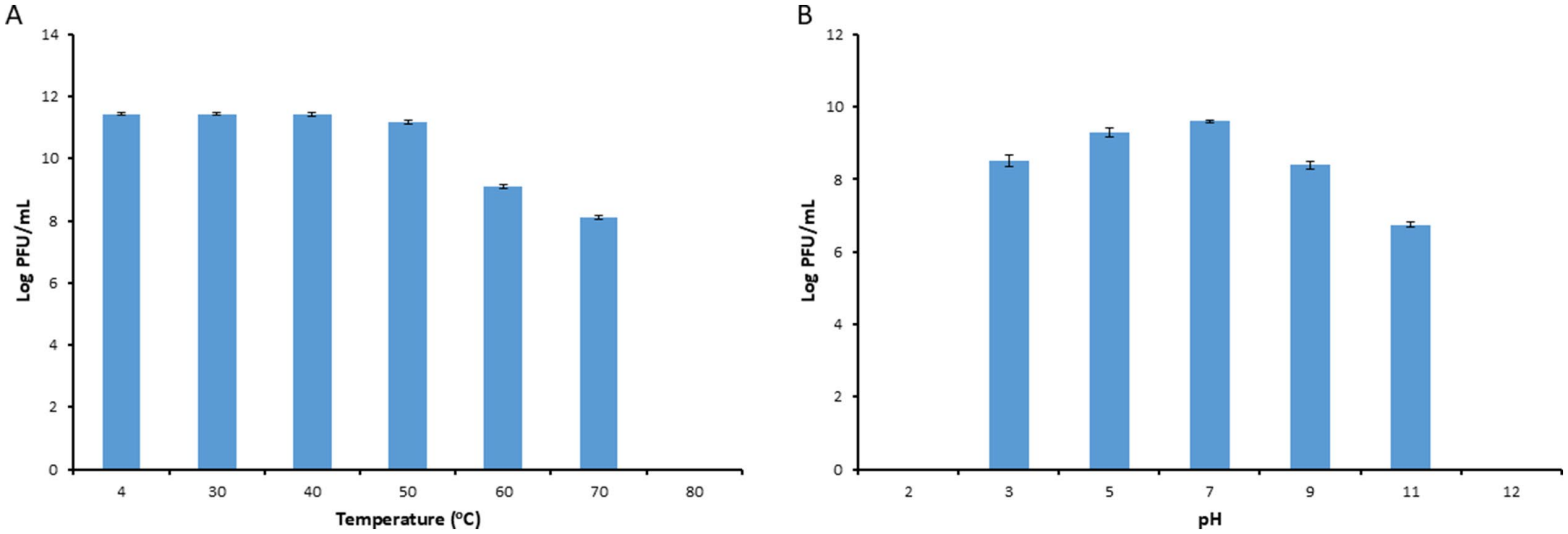
Stability of phage PbrM1EGY under different temperature **(A)** and pH **(B)** conditions. Phage PbrM1EGY (3 × 10^11^ PFU/mL) was incubated under each condition and its titer was estimated by plaque assay using *P. brasiliense* strain KMBR1 as a host 1 h after incubation. All values represent means of three determinations. Bars represent standard deviation.

The thermal stability test results indicated that the activity of phage PbrM1EGY was stable between 4–50°C for a period of 1 h, maintaining approximately 1.54 to 2.8 × 10^11^ PFU/mL at temperatures of 4, 30, 40 and 50°C. The phage, however, significantly reduced its viability after incubation for 1 h at 60 and 70°C, with its titers reduced to 1.29 × 10^9^ and 1.30 × 10^8^ PFU/mL respectively, and no phage was detected after incubation at 80°C for 1 h (Figure 4A).

Our pH stability assessment results indicated that PbrM1EGY was relatively stable at a pH range of 3 to 11, with an optimal pH of 5 to 7 since the phage titers remaining to be 2.1 × 10^9^ to 4 × 10^9^ PFU/mL (Figure 4B). At pH values of 3, 9 and 11, the phage titers were significantly reduced to 3.1 × 10^8^, 2.4 × 10^8^ and 5.5 × 10^6^ PFU/mL, respectively (Figure 4B). At pH values of 2 and 12, the phage was inactivated since no phage activity was detected (Figure 4B).

### 3.6 Stability of phage PbrM1EGY under different salt concentrations and UV exposure times

**Figure 5:**
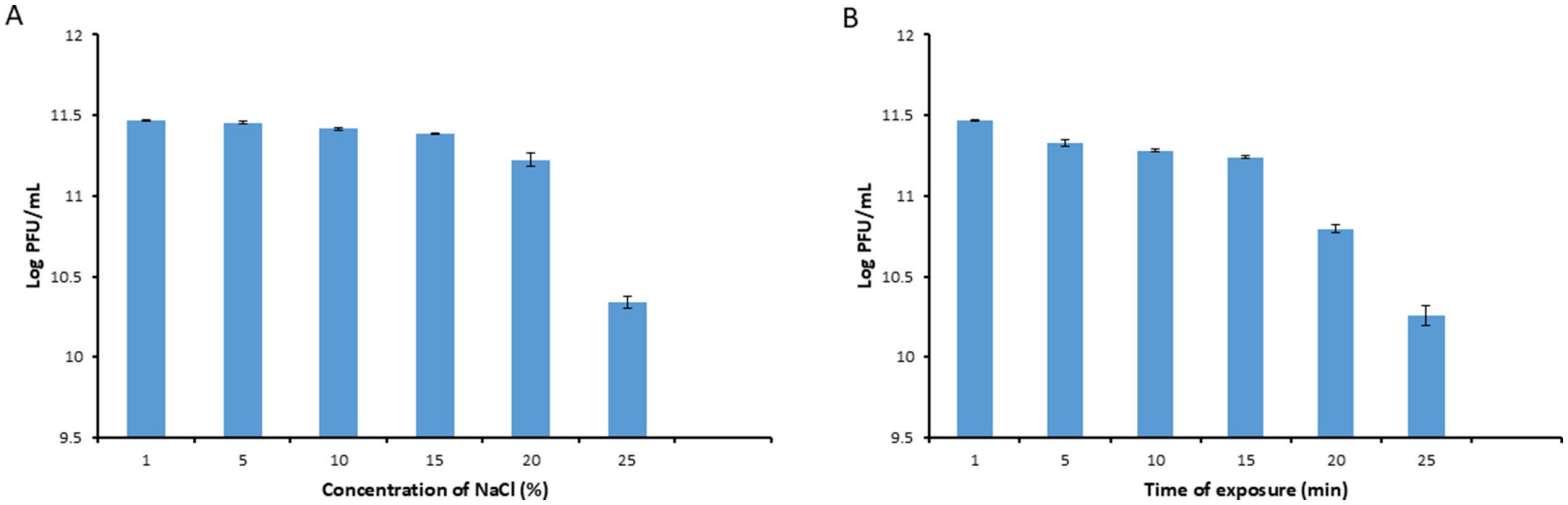
Stability of phage PbrM1EGY under different NaCl concentrations **(A)** and UV exposure times **(B)**. Phage PbrM1EGY (3 × 10^11^ PFU/mL) was incubated under each indicated condition and its titer was estimated by plaque assay using *P. brasiliense* strain KMBR1 as a host 1 h after incubation. All values represent means of three determinations. Bars represent standard deviation.

Phage PbrM1EGY was relatively stable after incubation for 1 h at NaCl concentrations ranging from 1 to 15%, since the phage titers remained to be over 2 × 10^11^ PFU/mL (Figure 5A). Its titer, however, was reduced after incubation at the salt concentration of 20% to 1.7 × 10^11^ PFU/mL, and further reduced to 2.2 × 10^9^ PFU/ml at salt concentration of 25% (Figure 5A).

As shown in Figure 5B, the titers of phage PbrM1EGY remained to be above 1 ×10^11^ PFU/mL after exposure to UV irradiation from 1 to 15 min (Figure 5B). Its titer, however, was reduced significantly after UV exposure for 20 and 25 min, to 6.3 × 10^10^ and 2 × 10^10^ PFU/mL, respectively, (Figure 5B).

### 3.7 Genomic characteristics of phage PbrM1EGY

**Figure 6:**
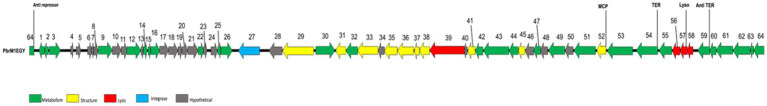
Genomic organization of phage PbrM1EGY. Colored arrows indicate the directions and categories of the genes. The color codes are: green, metabolism; yellow, structure; red, lysis proteins; blue: integrase; gray: hypothetical protein. Numbers are annotated ORFs. MCP, phage minor head protein; TER, terminase; Lyso, lysozyme; Anti TER, anti-terminator.

The genome of phage PbrM1EGY was determined to be 42,408 bp in length with a G + C content of 52%. The complete genome sequence was deposited into GenBank under the accession number PQ676537. When its whole genome was used as a query to search NCBI’s nucleotide collection by BLASTn, the phage exhibited only marginal similarity to Serratia phage vB_SspM_BZS1 (accession number MT843275.1 for its complete genome sequence) with 95.5% nucleotide identity over 16% of the genome. Sixty-four potential open reading frames (ORFs) with start codon of ATG or GTG were identified within the genome of PbrM1EGY (Figure 6; Supplementary Table S1). Information including their positions, best homologs and predicted functions are summarized in the Supplementary Table S1. Of the 64 ORFs, 43 had homologs in GenBank with predicted functions related to phage structure, DNA packaging, replication, transcription, integration, and lysis, while 21 were classified as hypothetical proteins (Supplementary Table S1).

### 3.8 Gene organization of phage PbrM1EGY

The annotated ORFs of phage PbrM1EGY can be classified into four distinct functional gene categories: metabolism (transcription and regulatory functions), structural components (including head and tail), lysis and integration, and hypothetical or other proteins (Figure 6; Supplementary Table S1).

#### 3.8.1 Metabolism

Twenty-eight ORFs were annotated to play a role in the phage’s metabolism (Figure 6; Supplementary Table S1). Of the 28 ORFs, ORF1 was predicted as a lambda phage CII family protein, ORFs 2, 3, and 62 as helix-turn-helix domain containing proteins, ORF 12 as a DNA adenine methylase, ORFs 14, 15, and 16 as HNH endonucleases, ORF 26 as a DNA methyltransferase, ORFs 54 and 55 as Phage terminases, ORF 59 as an antitermination protein, and ORF 64 as a phage anti-repressor protein.

#### 3.8.2 Structure

Ten ORFs were predicted to be involved in morphogenesis of phage PbrM1EGY (Figure 6; Supplementary Table S1). ORFs encoding phage capsid-related proteins included ORF 52 for putative phage minor head protein, and ORF 45 for putative head to tail adaptor. ORFs encoding phage tail-related proteins included ORF 41 for phage tail assembly, ORF 29 and 31 for tail fibers, ORFs 33, 37 and 38 for phage baseplate, ORF 36 as a phage protein, and ORF 35 for puncturing spike protein.

#### 3.8.3 Lysis and Integration

Four ORFs (39, 56, 57 and 58) were predicted to be involved in lysis of bacterial cells (Figure 6; Supplementary Table S1). ORF 39 was annotated to encode a lytic transglycosylase domain-containing protein, ORF 56 a lysis protein, ORF 57 a putative lysozyme, and ORF 58 a phage holin protein. ORF 27 of phage PbrM1EGY was predicted as a phage integrase that may mediate site-specific recombination between phage PbrM1EGY and its host bacterial host strain.

### 3.9 Effect of phage PbrM1EGY on *in vitro* growth of *Pectobacterium brasiliense*

**Figure 7:**
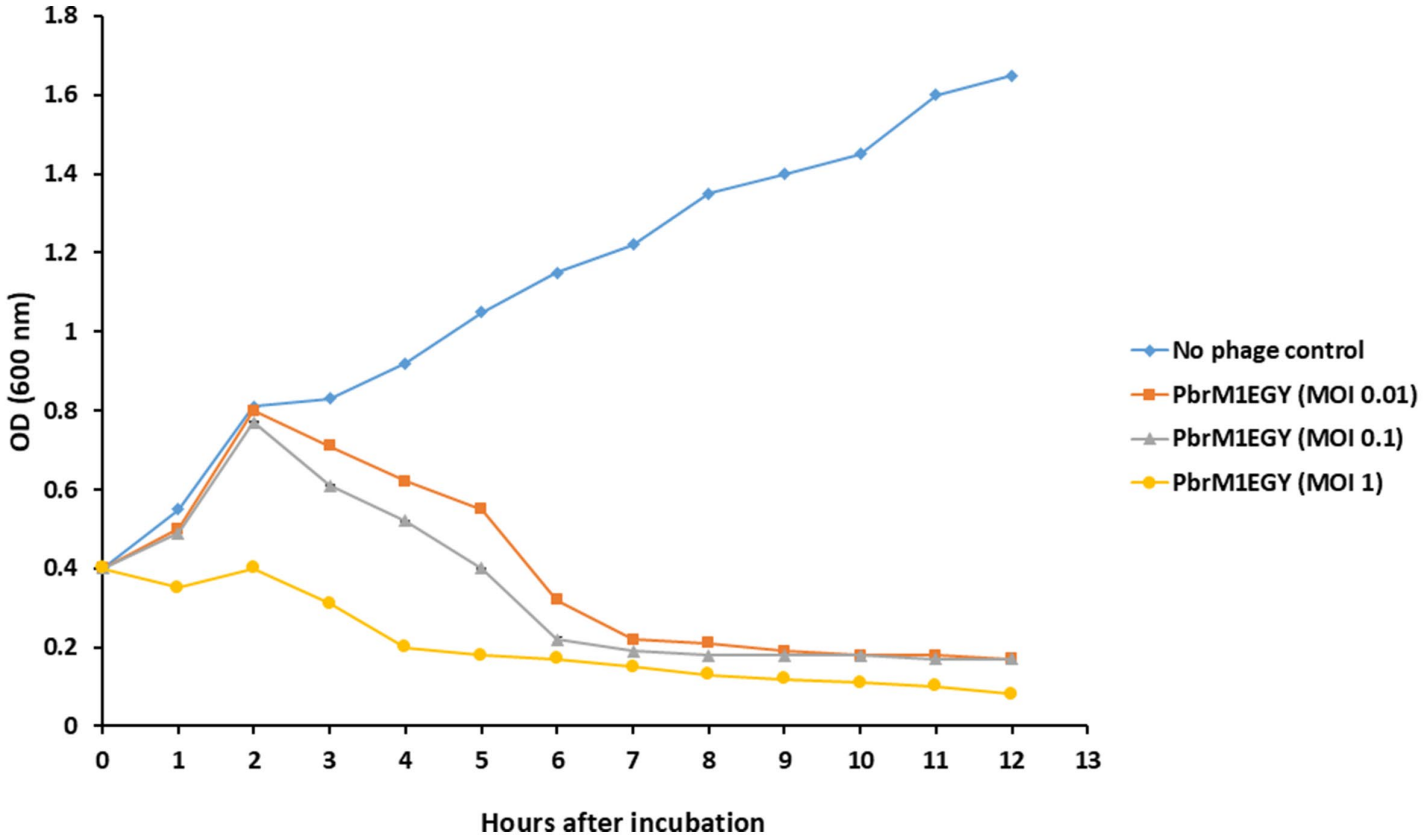
Effect of phage PbrM1EGY on *in vitro* growth of *P. brasiliense* at different MOIs (1, 0.1, and 0.01). Each value represents a mean of three replicates. Bars represent standard deviation.

When phage PbrM1EGY was added to *P. brasiliense* cells at MOIs of 1, 0.1 and 0.01, the phage significantly reduced the *in vitro* growth of *P. brasiliense* at all MOIs 6 h after incubation (Figure 7). When no phage was added to *P. brasiliense* cells, the bacterium grew from OD_600_ of 0.4 to 1.65 within 12 h of incubation, while adding the phage to the bacterium at MOI of 1 reduced the bacterial growth to OD_600_ of 0.17 within the first 5 h and to 0.08 within 12 h after incubation (Figure 7).

### 3.10 Phage PbrM1EGY prevented soft rot in pepper, cucumber, carrot, and potato and reduced the disease in onion in laboratory assays

**Figure 8:**
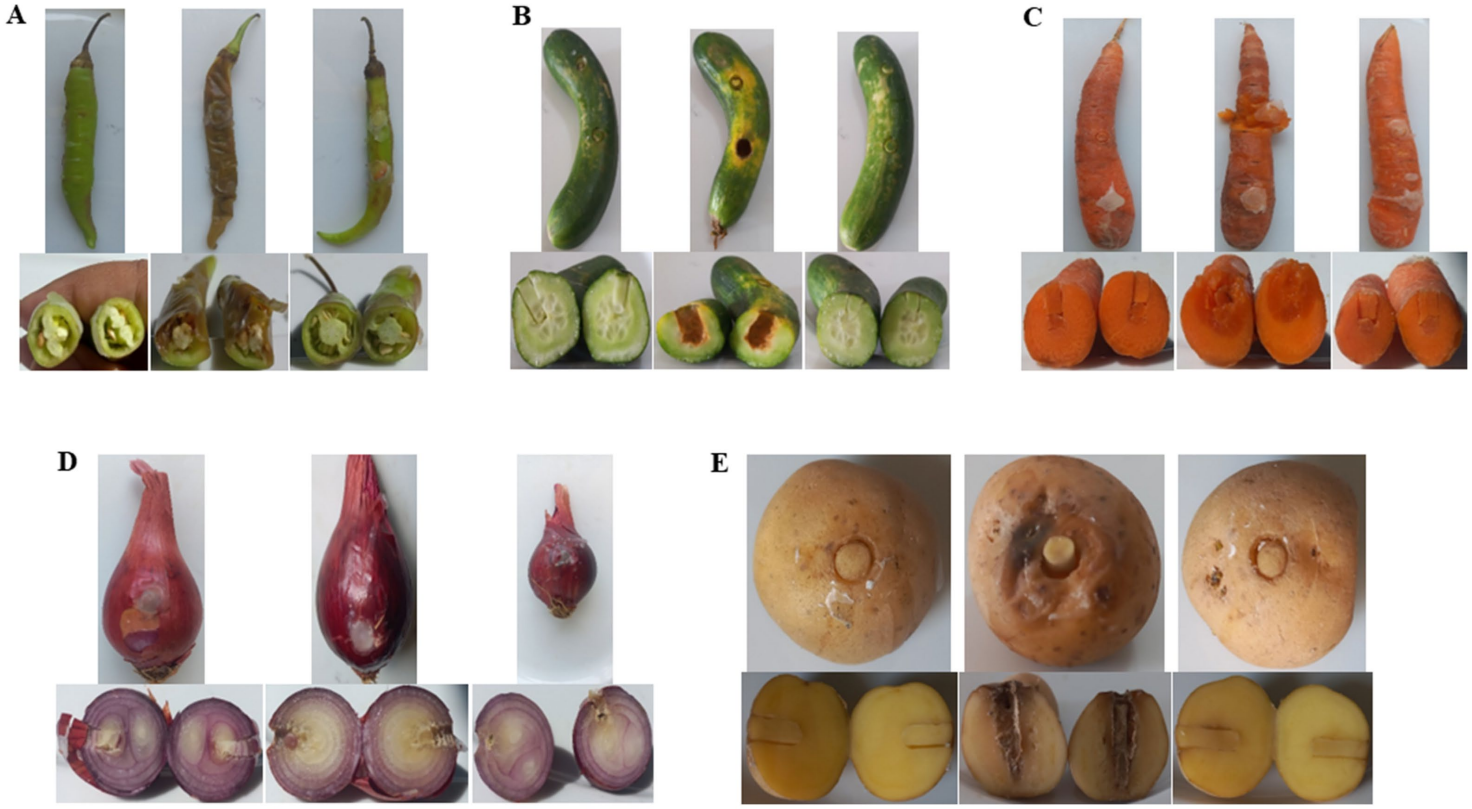
*In planta* bioassay demonstrating the protective effect of phage PbrM1EGY against *P. brasiliense* on pepper **(A)** and cucumber **(B)** fruits, carrot roots **(C)**, onion bulbs **(D)**, and potato tubers **(E)**. Each vegetable panel is accompanied by pictures showing the apprearance of the whole plant parts (top) and their cross sections (bottom) 72 h after inoculation with water (left), *P. brasiliense* (middle), or both *P. brasiliense* and phage PbrM1EGY (right).

**Figure 9:**
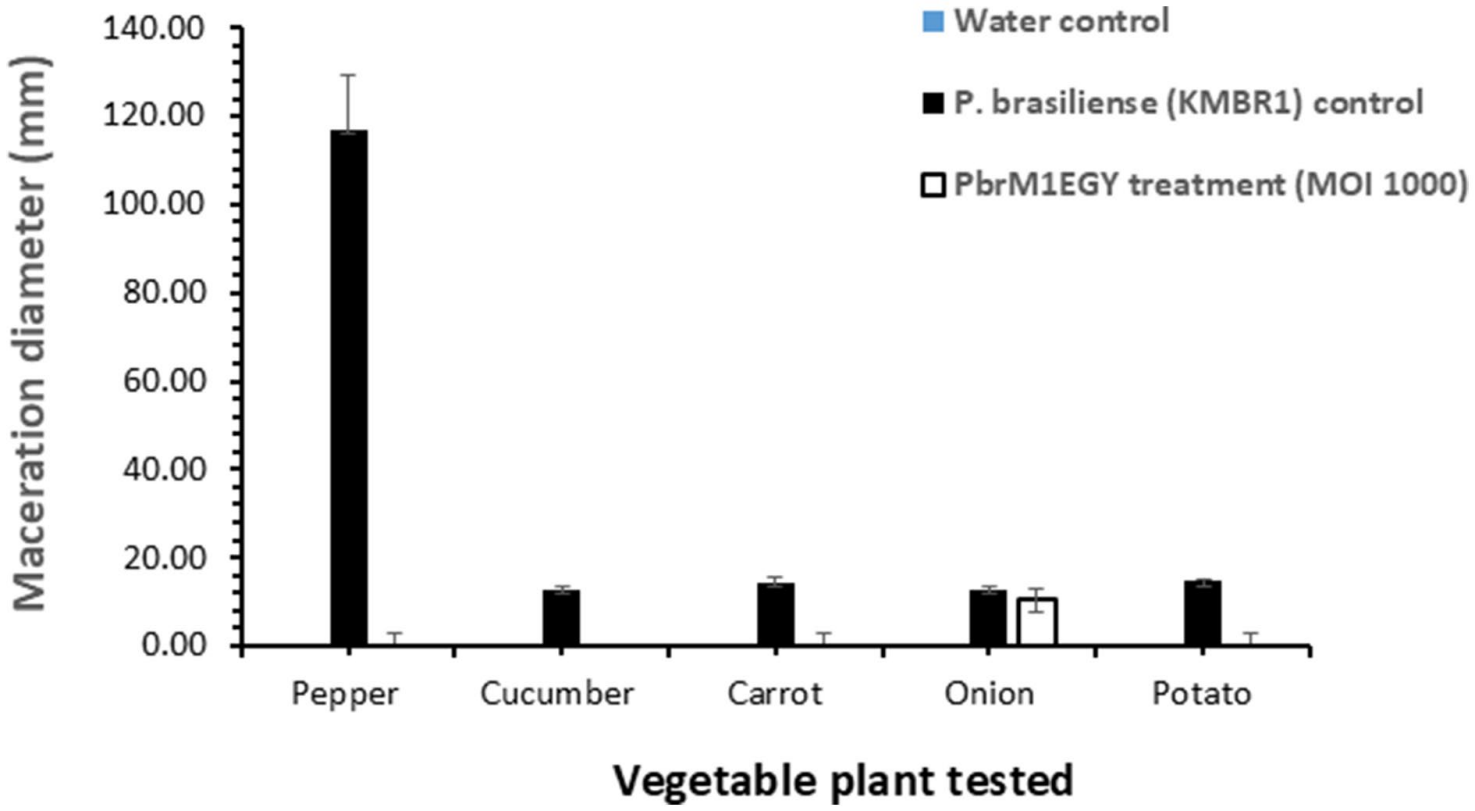
*In planta* application of phage PbrM1EGY prevented soft rot caused by *P. brasiliense* in pepper, cucumber, carrot, and potato, and reduced the disease in onion. The experiment was repeated twice, with each experiment containing five replicates per treatment per tested vegetable. Bars represent standard deviation. Significance was determined by Student’s t-test, comparing the *P. brasiliense* control and phage-treated groups separately for each vegetable type (*p* < 0.05).

Treatment with phage PbrM1EGY protected inoculated vegetable parts from tissue maceration caused by *P. brasiliense* (Figures 8, 9). The soft rot symptoms were completely suppressed in phage-treated pepper, cucumber, carrot, and potato (100% disease reduction), while the phage resulted in 18.1% reduction of tissue maceration in onion bulbs as measured by the diameter of rotten tissue in each treated plant part (Figures 8, 9). On the contrary, *P. brasiliense*-inoculated vegetable parts without phage treatment (pathogen control) displayed severe maceration symptoms 72 h after bacterial inoculation (Figures 8, 9). In *P. brasiliense*-inoculated pepper fruits, the maceration symptoms were so severe that the whole fruit was rotted (Figures 8, 9). Water treatments (water control) did not exhibit any soft rot symptoms (Figures 8, 9).

## 4 Discussion

Phages are considered a viable alternative antibacterial agent for controlling Plant diseses, including *Pectobacterium.* In a tuber assay using *P. atrosepticum* conducted under conditions resembling storage, Carstens et al. (2019) demonstrated that the application of a phage cocktail significantly reduced both the incidence and severity of the disease (Carstens et al., 2019). Moreover, in a tuber assay involving *P. atrosepticum* and *P. carotovorum* subsp. *carotovorum*, Zaczek-Moczydłowska et al. (2020) also showed that phages can reduce tissue maceration. Elhalag et al. (2024) demonstrated that in both a tuber maceration test and a greenhouse pot experiment, phages PcaP1EGY and PcaP2EGY provided significant protection against soft rot disease caused by *P. carotovorum* for both potato tubers and plants, compared to those inoculated with *P. carotovorum* without phage treatment.

*Pectobacterium brasiliense* is a bacterial plant pathogen found worldwide that causes soft rot in commercially important crops, including vegetables and ornamental plants. Symptoms of the disease can develop quickly, leading to the full maceration of plant tissue. In the past, only a few phages have been reported to infect *P. brasiliense* species (Oulghazi et al., 2021). Therefore, in this study, we characterized a novel lytic phage specific to *P. brasiliense*, which was isolated from Egyptian soil and evaluated as a potential biocontrol agent against *P. brasiliense*. Successful use of phages to protect potatoes from the Soft Rot *Pectobacteriaceae* (SRP) has been reported in both laboratory studies and field trials (Carstens et al., 2019; Voronina et al., 2019). The host range of phage PbrM1EGY was determined using a spot test, with additional confirmation provided by plaque assays. Phages are known to be highly specific, typically infecting only certain strains within a species or specific species within a given genus (Ross et al., 2016). Phage PbrM1EGY exhibited lytic activity exclusively against tested strains of *P. brasiliense*, demonstrating its high specificity. This is different from the two broad host range lytic phages vB_PcaP_PP2 and T-4 *Myoviridae* PM2, isolated from soil in soft rot-affected areas, since the two phages infected strains of both *P. carotovorum* and *P. brasiliense* species (Lim et al., 2015, 2017). In addition, Lukianova et al. (2020) reported the isolation of phages PP99 and PP101 from sewage water in a warehouse containing potatoes in Russia. Phage PP101 infected all strains of both *P. brasiliense* and *P. versatile*, whereas phage PP99 infected all strains of *P. brasiliense* and all strains of *P. versatile*, except for strain F100, which was resistant to phage PP99 (Lukianova et al., 2020).

Phage PbrM1EGY has a myovirus morphotype within the order Caudovirales with an icosahedral head of about 46.3 ± 3.9 nm in length, and forms clear medium-sized plaques (2–4 mm in diameter). The broad host range phages PP99 and PP101, however, form small (1–3 mm in diameter) plaques, normally indistinguishable from each other. Similar to phage PbrM1EGY, phage PP101 belongs to the family *Myoviridae* within the order Caudovirales, while phage PP99 has a typical *Podoviridae* morphology with an icosahedral capsid of 54 ± 3 nm in diameter and a short (approximately 10 nm) tail (Lukianova et al., 2020).

Phage PbrM1EGY has a larger burst size (749 ± 40 PFU/cell) and a shorter latent period (20 min) compared to *Pectobacterium* phage vB_PatM_CB7 of the genus *Certrevirus*, which has a smaller burst size (154 PFU/cell) and a longer latent period (55 min) (Buttimer et al., 2020). *Pectobacterium* phage P7_Pc also has a longer latent period of 125 min and a smaller burst size of 254 PFU/cell (Naligama and Halmillawewa, 2022). Similar to phage PbrM1EGY, *P. carotovorum*-infecting phage PcaP1EGY also exhibited a relatively large burst size of approximately 599 PFU/cell and a short latent period of 30 min (Elhalag et al., 2024). Phage growth characteristics, such as latent period, burst size, adsorption rate, can be significantly affected by bacterial growth and factors influencing bacterial growth rates, including the growth medium and other environmental conditions. Additionally, these phage growth parameters may vary depending on the specifics bacterial host species used in the experiment (Nabergoj et al., 2018).

Our study revealed that phage PbrM1EGY remained relatively stable within a pH range of 3 to 11. One of the key factors for optimal virus replication within a host bacterium is the ability of phages to withstand a wide range of pH levels and temperatures (Fister et al., 2016). The infectivity of most phages can be significantly affected by highly acidic or basic pH levels. Studies have shown that such conditions can denature phage proteins, ultimately compromising their viability (Hazem, 2002). Previous research also indicates that the majority of tailed phages are stable within a pH range of 5.0 to 9.0 (Smolarska et al., 2018; Fan et al., 2017).

The environmental factor found to have the most detrimental impact on the efficacy of biocontrol was UV light (Ignoffo and Garcia, 1994). The use of phages has faced certain challenges and limitations, particularly related to phage instability when exposed to UV irradiation. After exposure to UV radiation for 15 min, the titer of phage PbrM1EGY was decreased from 3 × 10^11^ to 1.73 × 10^11^ PFU/ml. In contrast, exposure to UV light for just 5 min completely inactivated *Pectobacterium* phages ϕA38 and ϕA41 (Smolarska et al., 2018). *P. versatile* phages Possum and Horatius did not survive after 10 min of UV exposure (Lukianova et al., 2022). Overall, our findings indicate that phage PbrM1EGY can withstand a wide range of environmental conditions, suggesting its potential for controlling soft-rot diseases in Egyptian fields.

Phage PbrM1EGY’s ability to endure NaCl concentrations from 1 to 25% suggests that it can tolerate high salt concentrations. Our findings align with a previous study by Smolarska et al. (2018), which reported no effect on phage survival when incubated in solutions containing 0.05, 0.5, and 5.0 M NaCl. Similarly, *P. versatile* phages Possum and Horatius was not affected by incubation in solutions containing 0.01, 0.1, 1, and 5.0 M NaCl (Lukianova et al., 2022).

Gill and Abedon (2003) suggested that several factors contribute to the success of phage therapy in plants, including the specific location and niche of the target bacteria, their density, the suitability of the solution for phage diffusion, and environmental conditions that support phage amplification. Phage PbrM1EGY treatment effectively reduced *P. brasiliense* cell growth in the liquid broth during the *in vitro* challenge assay, as revealed by the lysis kinetics of phage PbrM1EGY in Figure 7. In contrast, normal bacterial growth was observed in the absence of the phage (control), with bacterial cell concentration gradually increased over the incubation period. A key criterion for evaluating the potential use of the newly isolated phages in phage therapy is the bacterial reduction assay. Between three and 6 h post-infection, the lytic kinetics of phage PbrM1EGY showed a sharp decline in bacterial growth. However, at MOIs of 0.01 and 0.1, bacterial growth was slightly elevated, except at an MOI of 1.0. This result is considered a drawback of phage therapy, as previous studies suggest it may be due to the emergence of phage-resistant mutants following infection (Moldovan et al., 2007). To prevent or reduce the risk of resistance emergence during phage therapy, several strategies can be employed, including increasing phage concentrations, improving phage formulations and using phage cocktails. Increasing phage concentrations can lead to rapid bacterial killing, reduce the chances of bacterial adaptation, and overwhelm the bacteria’s defense mechanisms. Improving phage formulations helps protect phages from degradation, enhances delivery to the infection site and allows for sustained phage release. The use of phage cocktails - combinations of multiple phages targeting the same or different bacterial strains - lowers the likelihood of simultaneous resistance, as it is unlikely that the targeted bacteria will develop resistance to all the phages in the mixture at once. Before developing a phage cocktail, however, it is essential to determine the host range of each phage, as well as their genetic characteristics, application requirements, and effectiveness against the target pathogens. In Florida, for example, Balogh et al. (2008) treated citrus seedlings with a phage cocktail formulated in a protective skim-milk and observed a 50–60% reduction in the severity of bacterial spot and citrus canker disease. To manage *Xanthomonas axonopodis* pv. *allii*, the causal agent of Xanthomonas leaf blight in onions, more effectively and sustainably, phage treatments combined with copper compounds or plant activators were also examined (Lang et al., 2007). In addition, phage encapsulation in nanomaterials has shown promise in protecting phages from extreme pH and temperature conditions (Elsayed et al., 2024), enhancing their stability and efficacy under variable environmental conditions.

*Pectobacterium brasiliense* has the potential to cause rapid and severe pathogenicity in plants, leading to swift tissue decay and, in some cases, plant death (Queiroz et al., 2017). This study highlights its highly harmful nature, as evident from the food rot observed just 72 h after bacterial inoculation. This highly pathogenic bacterium has been shown to inflict significant damage on a variety of crops, including peppers (Gillis et al., 2017), tomatoes (Jaramillo et al., 2017), and potatoes (Marković et al., 2021).

The results of our *in planta* bioassay evaluating the protective activity of phage PbrM1EGY showed that *P. brasiliense* (pathogen control) infected the fruits, bulbs, roots or tubers of five crops from four families, including Amaryllidaceae (onion), Solanaceae (potato and pepper), Umbelliferaceae (carrot), and Cucurbitaceae (cucumber). This aligns with previous reports indicating that *P. brasiliense* infected 10 crops across four families (He et al., 2023). Our experiment further demonstrated that phage PbrM1EGY effectively protected onion, potato, pepper, carrot, and cucumber from soft rot. The limited effectiveness of phage PbrM1EGY in onion bulbs, resulting in only 18.1% reduction in tissue maceration compared to the 100% reduction observed in other tested vegetables, may be attributed to several factors. The natural antimicrobial compounds present in onions could trigger a different bacterial stress response, potentially reducing phage replication efficiency. Additionally, the layered and loosely structured anatomy of onion bulbs may hinder effective phage penetration and distribution. Furthermore, the biochemical composition of onions might favor bacterial proliferation, thereby diminishing the effectiveness of the phage treatment. Our findings are consistent with prior studies showing that the two lytic phages, *Podoviridae* PP99 and *Myoviridae* PP101, were highly specific against *P. brasiliense* and could serve as potential biocontrol agents (Lukianova et al., 2020).

## 5 Conclusion

A virulent phage PbrM1EGY was isolated from Egypt and characterized for its potential as a biocontrol agent. Morphological and genomic analyses revealed that the phage belongs to *Myoviridae* family. Phage PbrM1EGY exhibited a high burst size and demonstrated stability under various tested conditions, including exposure to ultraviolet light. At different MOIs, phage PbrM1EGY effectively inhibited the *in vitro* growth of *P. brasiliense* compared to the control group which received no phage treatment. Furthermore, treatment with phage PbrM1EGY significantly suppressed or reduced the development of soft rot symptoms in various parts of tested vegetables, including pepper and cucumber fruits, carrot roots (the edible taproot), onion bulbs and potato tubers inoculated with *P. brasiliense*.

## Data Availability

The datasets presented in this study are publicly available. This data can be found at: https://www.ncbi.nlm.nih.gov/genbank, accession number PP484882.

## Footnotes

^1^ http://vassarstats.net/anova1u.html
